# Special Issue on Nanoparticles in Nanobiotechnology and Nanomedicine

**DOI:** 10.3390/ijms26010267

**Published:** 2024-12-31

**Authors:** Costica Caizer

**Affiliations:** Department of Physics, Faculty of Physics, West University of Timisoara, Bv. V. Pârvan No. 4, 300223 Timisoara, Romania; costica.caizer@e-uvt.ro

In the present day [1], nanoparticles are of high theoretical and applicative interest; they have multiple applications in nanotechnology and nanomedicine due to their small size (nm to hundreds of nm), which gives them different properties to those of bulk materials. Modern nanobiotechnology enables the preparation and dispersion of nanoparticles in different environments, as well as their biofunctionalization, bioencapsulation, bioconjugation, biosurfactation, etc.; this renders them biocompatible with the biological environment in which they are applied and enables their manipulation for applications in the field of diagnostics, in therapy for various diseases, and in nanotheranostics, as well as in alternative cancer therapies.

This Special Issue aims to highlight current research results in this field in the form of articles, as well as the systematization of recent results in the form of reviews.

Thus, a study is presented [2] on the CRIF1 siRNA-encapsulated PLGA nanoparticles that reduced tumor growth in MCF-7 xenograft mice (PLGA: copolymer of poly(lactic-*co*-glycolic) acid; CRIF1: CR6-interacting factor 1). This is due to the inhibition of mitochondrial synthesis of the OXPHOS (oxidative phosphorylation) protein by CRIF1 deletion, which destroyed mitochondrial function, leading to increased levels of ROS (reactive oxygen species), as well as the induction of antitumor effects in MCF-7 breast cancer cells. Also, in Ref. [3], a fundamental study is presented on the influence of the protein corona (PC) on the surface of antibody-functionalized lipid liquid nanocapsules (LLNCs (nanoparticles consisting of olive oil that are 100–200 nm in diameter)) on interacting with cancer cells (breast cancer cell (SKBR3)) using the αHER2 antibody. It is shown that the PC surrounding the nanocarrier (LLNCs-αHER2-PC) allows for the specific recognition of HER2 receptors in cancer cells, although decreasing the uptake efficiency (up to 40%). This result is important in future targeted cancer therapy through the use of the nanobiotechnology regarding the encapsulation and biofunctionalization of biocompatible and biofunctionalized nanocarriers with antibodies for the targeted therapy of tumor cells without toxicity (not affecting healthy cells). Another development of targeted delivery to tumor cells based on selective absorption by certain receptors on the surface of the target cell is shown in Ref. [4]. Here, a fullerene C60 conjugate with polyvinylpyrrolidone (PVP) as a biocompatible biostructure, as well as folic acid (FA) as a targeting ligand (FA-PVP-C60) for tumor cells with an increased expression of folate receptors (FRs), was obtained. It was shown that FA-PVP-C60 nanobioconjugates do not show cytotoxicity in vitro up to a 200 μg/mol concentration; they are absorbed by HeLa cells (a cervical cancer cell line), which have a high level of expression of this receptor, and are absorbed less by A549 cells (a lung carcinoma cell line) with a low FR expression. In the same area, an efficient method was proposed for vault nanoparticle conjugation with finely adjustable amounts of antibodies and small molecules for use in drug targeted delivery with high efficacy (by optimized targeting) [5]. The amelioration of neuroinflammation and neuronal losses in mice exposed to an anticholinesterase organophosphate by using scL-2PAM nanocapsules (oxime encapsulated within a cationic liposome decorated with a single-chain antibody fragment (scFv) recognizing the transferrin receptor (TfR) [6]) was proposed in Ref. [7]. The scL-2PAM nanoformulation (nanocomplex) is designed to cross the blood–brain barrier (BBB).

The use of rare earth metal nanoparticles as therapeutic agents in the regeneration of tissues without toxicity is of real interest in current nanobiotechnology and nanomedicine. Thus, a study on the influence of the synthesis scheme and concentration of rare earth metal nanoparticles of cerium dioxide (CeO_2_) (nanocerium) on cellular viability and cytotoxicity, with the aim of using them in wound regeneration (in the regeneration of skin cell structures such as fibroblasts, mesenchymal stem cells, and keratinocytes), is presented in Ref. [8]. The experimental results presented by the authors show that the CeO_2_ nanoparticles of 3–5 nm in size can be used at up to high concentrations of 10^−4^–10^−3^ M without cellular toxicity. Similar results are obtained for slightly smaller nanoparticles of 2–3 nm, but with a slight increase in cellular toxicity. Thus, it is concluded that nanocerium nanoparticles of 3–5 nm in size would be the best for use as therapeutic agents in the possible regeneration of skin cell structures without toxicity (the percentage of dead cells does not exceed 5%). In the same area of interest in nanobiotechnology and nanomedicine are the results presented in Ref. [9] regarding possible infection-free wound healing by using the biocompatible alginate gels incorporating silver (S) and tannylated calcium peroxide (CaO_2_@TA) spherical nanoparticles (obtained via adapted microfluidic and precipitation synthesis methods). The results obtained showed a synergistic antimicrobial effect between silver and tannylated calcium peroxide nanoparticles by oxygen release for the treatment of chronic wounds without infections. The test results showed an impressive antibiofilm efficiency against the *S. aureus* bacterial strain [9]. Another nanocomposite material based on rare earth magnetic nanoparticles with gadolinium (Gd)—the BaGdF5:Tb^3+^ nanocomposite, respectively—was synthesized using a single-stage microfluidic synthesis route, and was subsequently characterized and had its biodistribution studied in order for its use in modern nanobiotechnology as an efficient contrasting agent in NMR (nuclear magnetic resonance) and X-ray micro-CT (computer tomography), and a promising candidate for X-ray photodynamic therapy [10]. The proposed synthesis method allows us to obtain single-phase and monodisperse BaGd_1−x_F5:Tb^3+^ nanoparticles with a mean nanoparticle size of 7–9 nm and a hydrodynamic radius of approx. 22 nm. This opens up new pathways for nanotheranostic applications via using the photosensitive aqueous solution. Furthermore, the BaGd_1−x_F5:Tb^3+^@Rose Bengal conjugates obtained using this synthesis technique in vitro lead to the moderation of cytotoxicity and good cellular uptake, opening up opportunities for nanotheranostic applications in nanomedicine.

An interesting communication of current interest in modern nanomedicine regarding the possible toxicity produced by the use of gold nanoparticles (AuNPs) on direct DNA damage and oxidative DNA damage through reactive oxygen species (ROS) activation is presented in Ref. [11]. This study took into account the size and chemical coupling of Au nanoparticles, showing that 10, 22, and 39 nm AuNPs caused rapid (within minutes) and direct damage to DNA molecules without the involvement of ROS. This result, in addition to clarifying the controversy regarding ROS production, is very important in various areas of advanced nanomedicine, such as alternative cancer treatment or viral therapy (including COVID-19). In addition, Ref. [12] presents a study harvesting the power of the green synthesis of biocompatible gold nanoparticles (bAuNPs) (approx. spherical and ~30–140 nm in diameter) for different physicochemical conditions of synthesis, with the advantage of increasing the efficiency of biosynthesis compared to conventional chemical synthesis; cellular uptake; and reducing cytotoxicity, with a specific emphasis on their potential application in nano-oncology as effective antitumor target agents in prostate cancer cell lines; negligible effects are seen on non-neoplastic (HPEpiC) cells, significantly impacting on neoplastic (PC3) epithelial cell lines.

Furthermore, the use of ferrimagnetic nanoparticles [13,14] in superparamagnetic hyperthermia (SPMHT) [15] for an alternative therapy for human epidermoid squamous carcinoma (A431) in vitro is observed with a high efficacy on tumor cells (up to 83%) and without toxicity on healthy cells (keratinocytes (HaCaT) cells) (the cell viability of approx. 100%); this study is presented in Ref. [16]. Here, the ferrimagnetic magnetite nanoparticles (Fe_3_O_4_) were bioconjugated with hydroxypropyl-gamma-cyclodextrins (HP-γ-CDs) via the biocompatible polyacrylic acid (PAA) biopolymer, forming perfectly biocompatible Fe_3_O_4_-PAA-(HP-γ-CDs) nanobioconjugates. Fe_3_O_4_ nanoparticles (decorated with cyclodextrins) with an average diameter of ~16 nm were used in therapy at concentrations of 1, 5, and 10 mg/mL nanoparticles in cell suspension. Also, the duration of therapy was 30 min. at a temperature of ~43 °C, and magnetic fields of 168, 208, and 370 G (depending on the concentration of the magnetic nanoparticles) with a frequency of 312.4 kHz were used.

In addition to research articles and a communication, this Special Issue presents an extensive and well-documented review of different nanoformulations in pharmaceutical and biomedical applications, as well as green synthesis perspectives, for the design of new nanoformulations with therapeutic efficiency and their impact in relation to modern nanobiotechnology [17]. Thus, nanoformulations are widely used in pharmacy and nanomedicine due to their advantages that exceed conventional formulations and that allow improved solubility, bioavailability, targeted drug delivery, controlled release, and reduced toxicity [17].

In conclusion, nanoparticles remain of huge interest and high impact in current nanobiotechnology and nanomedicine due to the new potential applications still unexplored; therefore, research in this field continues to develop explosively at present, both fundamentally and applied (theoretical and experimental).
